# Biomechanical behavior of three different types of internal tapered connections after cyclic and static loading tests: experimental in vitro

**DOI:** 10.1186/s40729-020-00228-4

**Published:** 2020-07-21

**Authors:** Marcos Boaventura de Moura, Karine Regina Tolesano Loureiro, Livia Bonjardim Lima, Christian Felippi, Paulo Cézar Simamoto Júnior

**Affiliations:** 1 Department of Occlusion, Fixed Prosthodontics and Dental Materials, School of Dentistry, Federal University of Uberlandia, Av. Pará 1720, Bloco 4LB, sala 39, Uberlandia, MG 38405-902 Brazil; 2 Department of Mechanical Engineering, University Federal of Paraná, Curitiba, PR Brazil

**Keywords:** Biomechanics, Dental implants, Torque

## Abstract

**Abstract:**

**Background:**

In the long-term success of a dental implant, the reliability and stability of the implant-abutment interface are important. Studies of maximum force of dental implants with different loading values have been used. This study aims to evaluate the influence of the oblique cyclic loading on the maximum force supported in one-piece and two-piece abutments installed on internal tapered implants.

**Findings:**

Sixty implants and sixty prosthetic abutments were divided into six groups (*n* = 10): G1 and G2 (two-piece abutments with 16°), G3 and G4 (two-piece abutments with 11.5°), and G5 and G6 (one-piece abutments with 11.5°). A 2-Hz cyclic loading was applied to specimens of G2, G4, and G6, with a number of cycles of 2,400,000. All specimens were inclined by 30° from the vertical axis, and a vertical loading was applied over the tapered connections (ISO 14801). Then, the maximum force was tested by applying a static compression load on the specimens of the 6 groups tested (30°) at a rate of 0.5 mm/s. Statistical analysis was performed using the Shapiro-Wilk (*p* > 0.05) and Levene (*p* = 0.789) tests to determine if the data presented homoscedasticity and the Tukey test for multiple comparisons. Tukey test showed that the maximum force supported by G1 and G2 was not affected by the cyclic load, while in G3 and G4 it decreased significantly when subjected to the cyclic load. The G5 and G6 had a significant increase in maximum force supported when subjected to cyclic load.

**Conclusions:**

Cyclic loading influenced the maximum force supported of G4 and G6 but did not influence G2.

## Introduction

Several modifications in implant-abutment design have been made since the 1990s. The screws material and the coefficient of friction between the coupled surfaces were made to reduce the complications of the connection [1]. The mechanical complications of external connections remain a concern in the implant community. To overcome the connection problems, a new concept of internal connection has been developed [2]. The internal connection has mechanical advantages, such as drastic reduction of screw fractures, distancing the occlusal forces deep into the implant, and protecting the screw from overload [3]. In addition, deep joints in internal connections are more prone to withstand bending forces than the flat joints of external connections. The internal tapered connections appear to be more resistant to screw loosening, abutment movement, and loss of torque, thus being more resistant to fatigue loading [3, 4].

In the internal tapered implant-abutment joints, the fixation and stability are conferred by the frictional resistance resulting from the contact between the tapered parts of the tapered abutment and implant coupling, not being a function of the screw. The application of axial compressive forces causes the increasing of the frictional resistance resulting from the contact of the tapered coupling parts [5]. Mathematical formulas and finite element (FE) models have shown that more than 86% of the tightening torque and more than 98% of the relaxation torque are balanced by the tapered junction of these systems [5]. The application of the occlusal load is a factor that can lead to loosening of the retention screw and abutment movement [6]. Therefore, in vitro mechanical tests have been successfully used for the application of cyclic loads to simulate masticatory forces [4, 7–11].

The geometry of the implant-abutment interface seems to be an influence factor for the transmission of stress around the implant [12]. The use of a tapered connection and abutment with integrated screw (two-piece) is becoming more popular, where the screw and a tapered adjustment are used simultaneously to provide mechanical stability. This type of joint offers high resistance to screw loosening torques and abutment [12].

Several studies have tested dental implants using static loading, while others have used cyclic loading. Failures in the implant-abutment complex usually occur in the application of repeated cyclic loads [13]. Most literature does not mention fatigue as a complex failure mode, influenced by structural design, material properties, and environmental effects. Most of the cycle tests focused on the design of the implant-abutment interface, and only a few studies have addressed the effect of implant diameter on fatigue performance [14]. Although repeated loads may be considered a preponderant cause of failure of dental implants, systematic and qualitative data are quite scarce [14].

The stability of the implant-abutment assembly is critical to the long-term success of implant-supported restorations [8, 9, 11, 12]. Internal tapered connections are known to have good biomechanical stability [11], but further studies remain interesting to be performed, since configurations of different geometries, both implant and abutments, are often launched in the market. Therefore, it is important to study some aspects of these new implant-abutment type configurations, such as biomechanical resistance and stress distribution.

This study evaluated the influence of the oblique cyclic loads on the result of the static loading test (maximum force) in one-piece and two-piece abutments installed on internal tapered implants. Three types of abutments with internal tapered connections were evaluated: A solid one-piece abutment with an apical threaded portion, with internal angle of 11.5° and two two-piece abutments with a transfixed screw, one of them with internal angle of 11.5° and another of internal angle of 16°. The null hypothesis tested was as follows: there is no difference in maximum force supported between implant-abutment assemblies.

## Materials and methods

### Specimens preparation

**Fig. 1:**
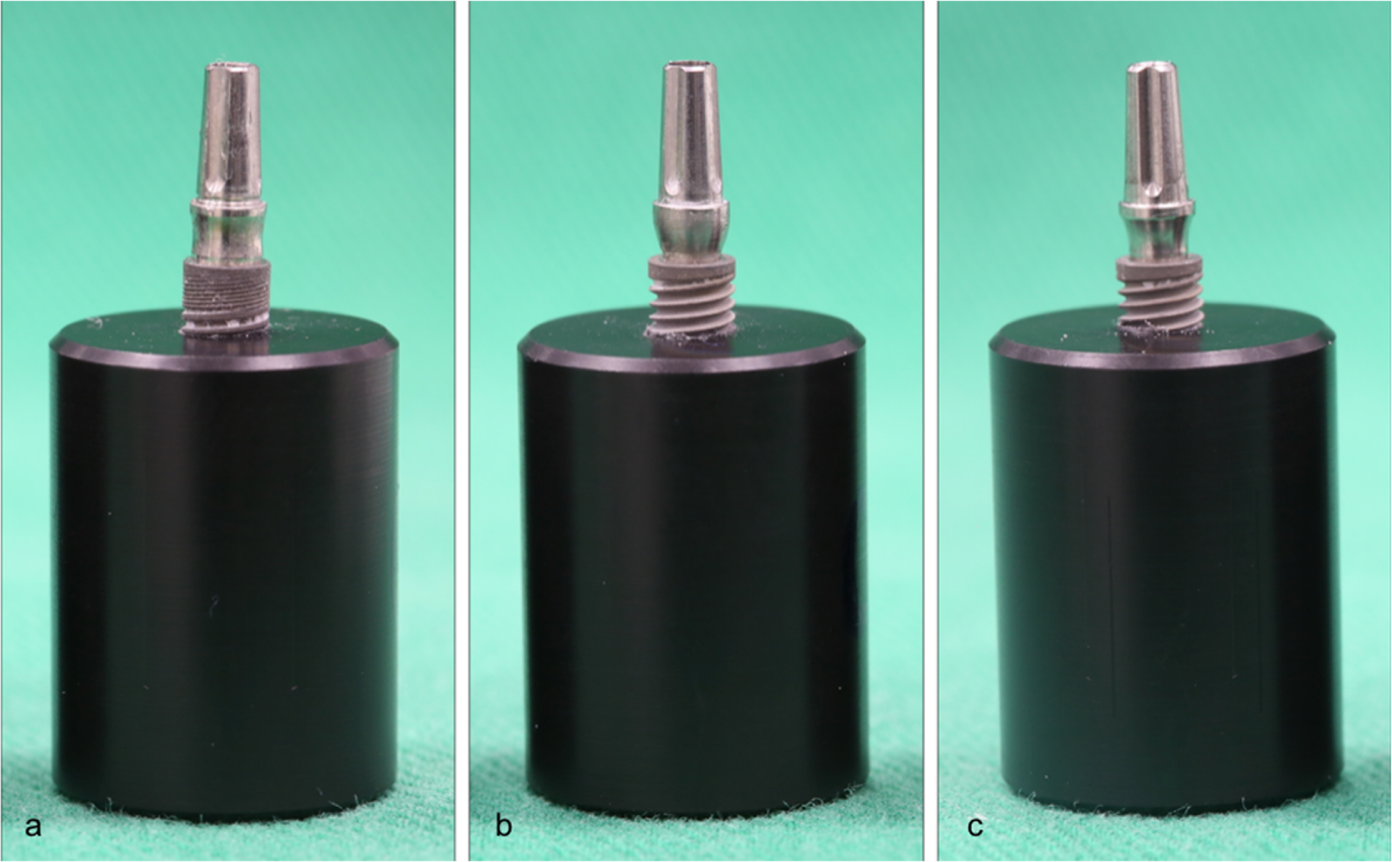
Types of implant-abutment connections used in the study. **a** G1 and G2, two-piece abutment with 16°. **b** G3 and G4, two-piece abutment with 11.5°. **c** G5 and G6, one-piece abutment with 11.5°

**Fig. 2:**
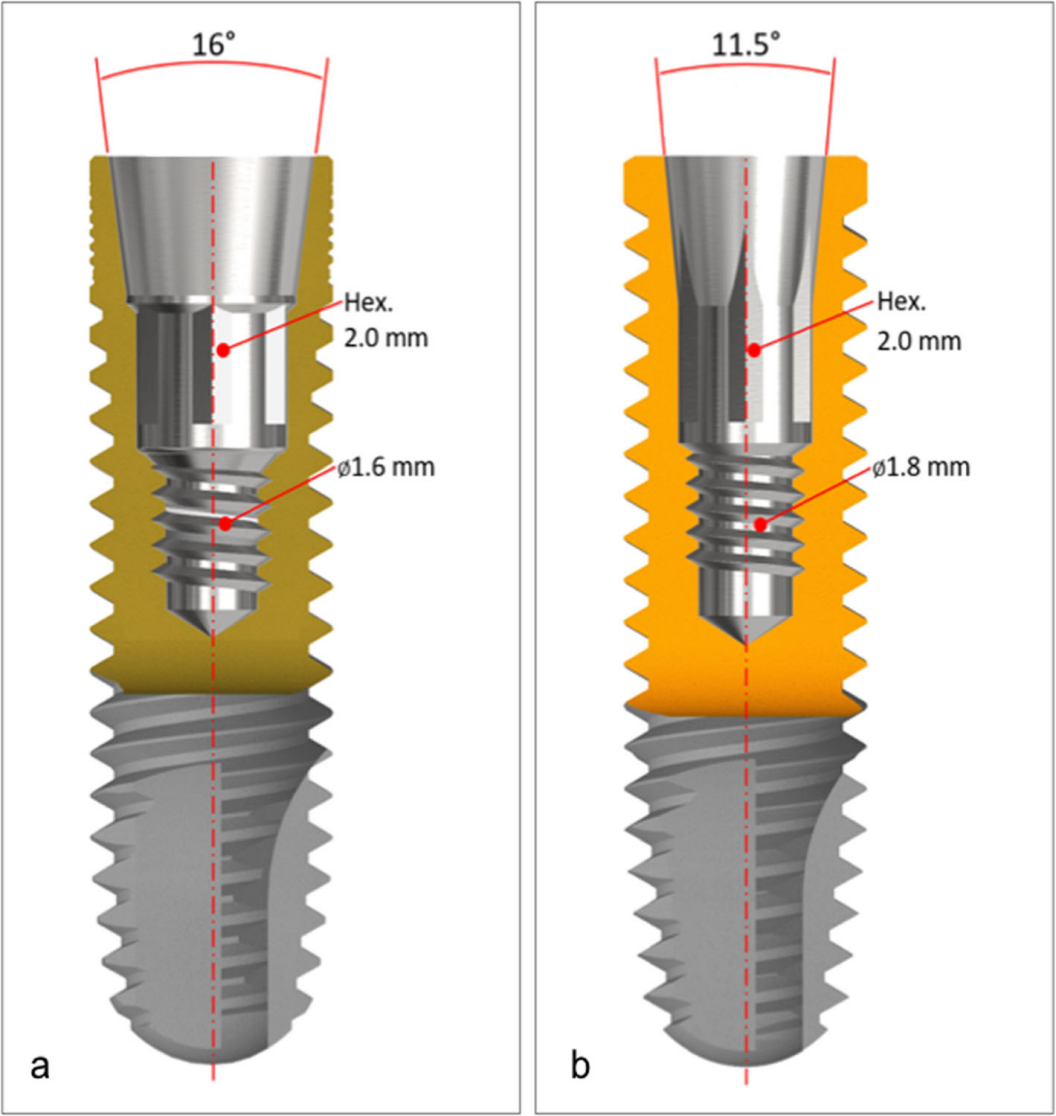
Different internal angulations of tapered implants used. **a** G1 and G2, Titamax GM implant (16°). **b** G3 to G6, Titamax Cortical CM implant (11.5°)

Sixty implants with a Morse taper design and 60 prosthetic abutments (Neodent, Curitiba, PR, Brazil) were connected using three different tightening forces: 20 Ncm (G1 and G2), 15 Ncm (G3 and G4), and 32 Ncm (G5 and G6). Each experimental group was formed by ten implants and ten abutments (*n* = 10) for a total of 60 specimens (*n* = 60) (Table 1) (Figs. 1 and 2).

**Table 1:** Test groups and conditions

Group	Implant	Abutment	Connection type/index/internal angle
1	Titamax GM; Ø3.75/13 mm	Universal Abutment GM exact Ø3.3/6/2.5 mm (two-piece)	Internal tapered/internal hexagon/16°
2	Titamax GM; Ø3.75/13 mm	Universal Abutment GM exact Ø3.3/6/2.5 mm (two-piece)	Internal tapered/internal hexagon/16°
3	Titamax Cortical CM; Ø3.75/13 mm	Universal Abutment CM through screw Ø3.3/6/2.5 mm (two-piece)	Internal tapered/without index/11.5°
4	Titamax Cortical CM; Ø3.75/13 mm	Universal Abutment CM through screw Ø3.3/6/2.5 mm (two-piece)	Internal tapered/without index/11.5°
5	Titamax Cortical CM; Ø3.75/13 mm	Universal Abutment CM Ø3.3/6/2.5 mm (one-piece)	Internal tapered/without index/11.5°
6	Titamax Cortical CM; Ø3.75/13 mm	Universal Abutment CM Ø3.3/6/2.5 mm (one-piece)	Internal tapered/without index/11.5°

All implants were placed in a cylindrical block made of polyacetal, leaving 3 mm of exposure of these implants, as recommended by ISO 14801 (Fig. 1). A digital prosthetic ratchet (TQ-680, Instrutherm) was used to tightening the prosthetic abutments to the implants.

### Dynamic loading test

**Fig. 3:**
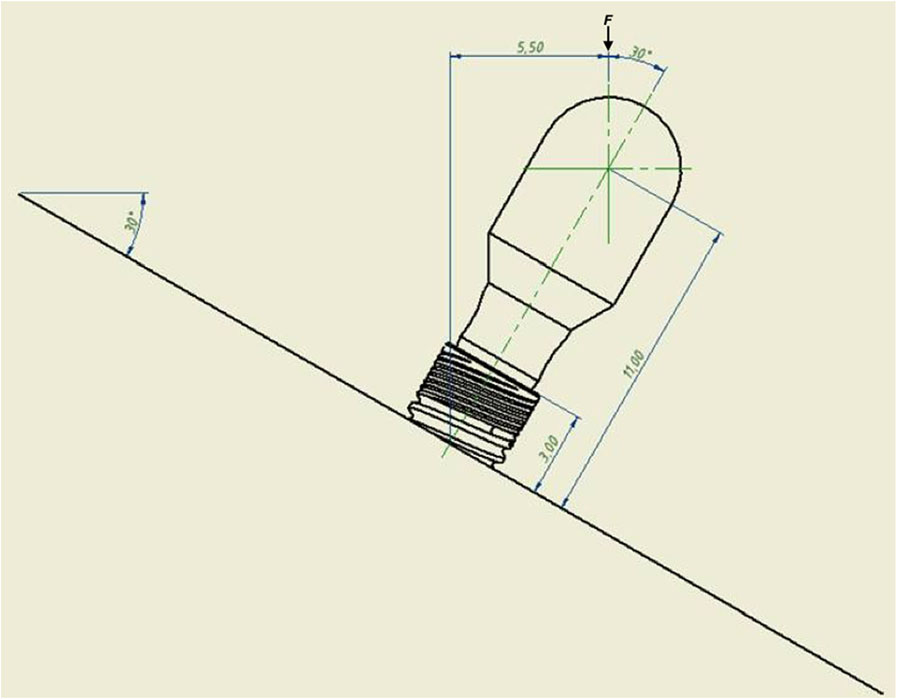
Schematic of dynamic loading test

A cyclic loading was applied to 30 specimens (G2, G4, and G6) using a mechanical cycler for specimens sliding fatigue tests (Electropuls E1000, Instron). To simulate a worst-case condition, specimens were inclined at 30° from the vertical axis for vertical load application, combining bending and torqueing moments on the internal tapered connection. On the cementable part of the abutments was used a semispherical rigid body, the center of which coincided with the center of the free longitudinal axis and was anchored at 11.0 ± 0.5 mm (measured on a line parallel to the longitudinal axis of the implant). A force load of approximately 50 N averaged over each implant-abutment assembly was applied at a frequency of 2 Hz and a temperature of 37 °C ± 1 °C. An amount of 2,400,000 cycles were applied on the specimens (Fig. 3).

### Static loading test

**Fig. 4:**
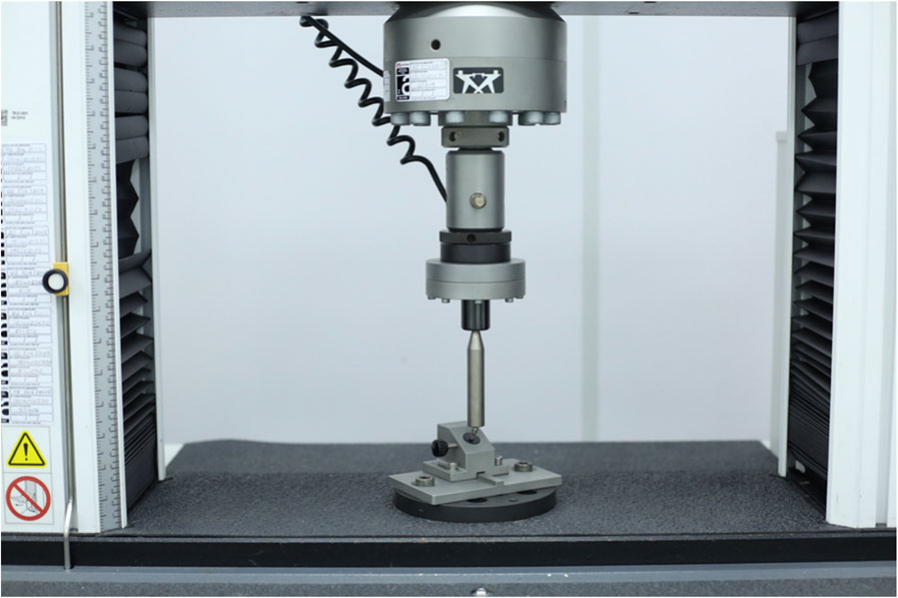
Static loading specimen setup simulating a worst-case condition

For the application of the static compression loads in the 60 implant-abutment assemblies in an electrodynamic test system (ElectroPuls E3000, Instron), a semispherical rigid body was used on the abutments and a 30° angulation of the specimens (Fig. 4). A flexural load was applied to the implant-abutment assemblies at a rate of 0.5 mm/s. The bending load acquired by the load cell was plotted on a load versus displacement curve. A computer associated with the machine was programmed to obtain the mechanical behavior of the specimen using a load cell and a load sensor. The test machine was programmed to stop the force test process for a greater displacement of 5.0 mm or an abrupt decrease in the strength of the tested material.

**Fig. 5:**
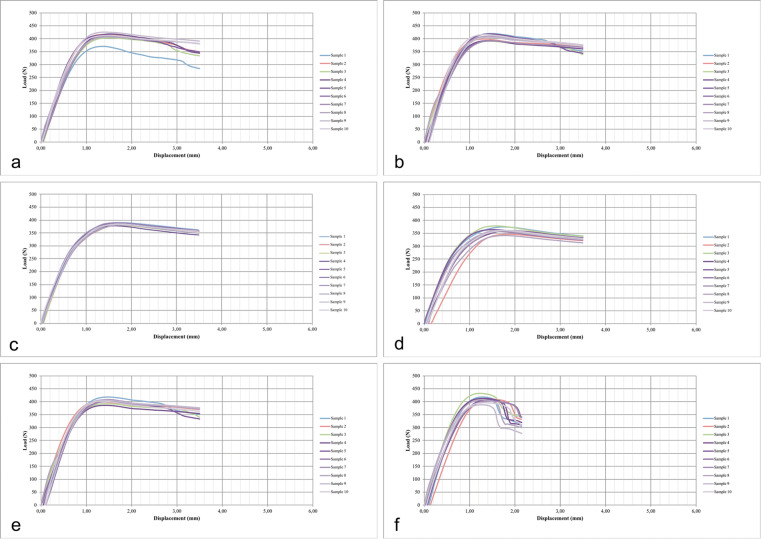
Graphic curves generated (load [N] × displacement [mm]) show the elastic limit behavior of the 6 groups to the different loading. **a** G1. **b** G2. **c** G3. **d** G4. **e** G5. **f** G6. The different colors of the lines represent each tested specimen for each group

The elastic limit (Fle) was represented by the limit point before the plastic or in irreversible limits. Titanium is a material that presents no clear evidence of the exact point between the plastic and elastic limits. The value of the load corresponds to the elastic limit of the material to be tested [15]. In this study, a flexion test was performed by means of static loading on the implant-abutment assemblies. The data curves were generated showing the behavior of the elastic limit for the different loading situations (Fig. 5). The elastic limit values (Fle) were extracted from the measured data using the transition point between linear and non-linear curves generated from bending tests. The Shapiro-Wilk (*p* > 0.05) and Levene (*p* = 0.789) tests were used to determine whether the cyclic loading application affected the maximum force supported by different implant-abutment assemblies subjected to static loading. For the multiple comparisons between the groups, Tukey test was used. The statistical calculations were conducted in the SPSS 23 program (SPSS Inc., Chicago, IL, EUA), adopting a significance level of 5%.

## Results

No specimen failed after cyclic loading. This study did not evaluate screw loosening after cyclic loading because the specimens were subjected to static loading in sequence to assess the maximum force of each system. The averages and standard deviations of the maximum force (N) after static loading of the implant-abutment assemblies studied are described in Table 2.

**Table 2:** Means and standard deviations of the maximum force (N) supported by implant-abutment assemblies submitted or not to cyclic loading

Groups	Without cyclic loading	Groups	With cyclic loading
G1	408.28 Aa; (15.09)	G2	404.57 Aa; (11.32)
G3	386.01 Ca; (8.29)	G4	360.88 Bb; (11.18)
G5	402.34 Bb; (8.79)	G6	408.65 Aa; (11.97)

Averages followed by distinct capital letters indicate a statistically significant difference between assemblies, considering separately submitted and unloaded specimens (comparisons within each column). Means followed by distinct lowercase letters indicate a statistically significant difference between submitted and unloaded specimens, considering each assembly separately (comparisons within each line)

When there was no cyclic loading, the (G1) implant-abutment assembly had maximum force values significantly higher than those found with one-piece abutments (G5) which, in turn, withstood a significantly higher force than that verified for the with two-piece abutments (G3) (Table 2). On the other hand, when there was cyclic loading, although the implant with two-piece abutments (G4) also revealed the lowest values ​of maximum force, no statistically significant difference was found between the implant-abutment assemblies (G2) and one-piece (G6) (Table 2).

When the interaction between the implant-abutment assemblies (implant system) and the cyclic loading test was evaluated, the Tukey test revealed that for the G1 and G2, the maximum force was not significantly affected by cyclic loading (Table 2). As for the two-piece abutments (G3 and G4), the maximum force was significantly lower when the assemblies were subjected to cyclic loading. In contrast, for the one-piece abutments (G5 and G6), cyclic loading caused a statistically significant increase in maximum force (Table 2).

## Discussion

The results of this study do not support the acceptance of the null hypothesis tested. There was a difference in the maximum force supported between the different groups tested. Tukey’s test revealed that for the G1 and G2, the maximum force was not influenced by the cyclic load (Table 2). However, for the two-piece abutments (G3 and G4), the maximum force was significantly lower when they were subjected to cyclic loads. In contrast, the one-piece abutments (G5 and G6), the application of cyclic loading caused a statistically significant increase in maximum force (Table 2). Even with different results for maximum force supported, the final values ​were high. In a clinical situation, all connections would support masticatory forces, except G3 and G4 abutments for molars [16, 17]. Therefore, the dentist can choose which connection suits him best.

The tapered interlock between the tapered surfaces of the prosthetic abutment and the implant is expected to firmly secure the two components together, thus minimizing the importance of screw preload [16]. Therefore, the implant-abutment assemblies of G2, G4, and G6 could be tested by static loading without the need to verify the torques. The number of masticatory cycles simulated approximately 10 years of function [18], which is enough to evaluate the behavior of the abutments. Considering that the ideal is the abutment to be removed only if necessary, this shows that after a long period of simulated cyclic loading, there was no release and/or fracture of screws and no fracture of abutments [11]. However, the two-piece abutments may be more advantageous to the dentist due to their reversibility, as they have the screw that can be loosened more easily. On the other hand, the abutment of a part would be more difficult to remove in a situation of replacement of the prosthesis. Therefore, G1 and G2 would be the best option, since they supported more load compared to the other two-piece abutments.

When a specimen is subjected to any kind of loading, stress is generated. When these loads exceed the material production limit, implant failure may occur in clinical situations [15, 19]. The stress concentration resulting from occlusal forces on the prosthesis that dissipate to the implant/bone interface can lead to microfractures and bone loss around the implants. This factor can also cause implant mobility and fracture [19]. The magnitude of the stresses, the type of implant, and the amount of bone remaining will determine the tissue response. Bone loss caused by overload can probably occur in the crest region surrounding the implants and could favor implant fractures [19]. The test performed in this study simulated a similar loading situation, where the implants were tested with 3 mm exposure. The best aesthetic results would occur in the 11.5° connections, since they have the largest diameter implant walls and, consequently, a larger platform *switching*, which would favor a better gingival behavior [19].

The design of the internal joints provides a more homogeneous stress distribution around the implants, which will lead to decreased bone crest deformation [15, 20]. In this study, the maximum force of the implant was analyzed in a situation of maximum effort with flexion load applied on the abutment. The direction of load applied on the 30° angled specimens was chosen to simulate chewing action, characterized by interference, occlusal trauma, bruxism, and inclined implants. The type of failure that occurred in the connections was different and may be dependent on the angle or abutment. In two-piece connections, screw elongation and greater tension in the implant walls occur, while in one-piece abutments, fractures occur in the region of the first thread. However, the force resisted by the three connections is greater than that recommended by other authors [21]. Therefore, the three connections showed good results, and the choice is up to the dentist.

Acceptable chewing load values ​are controversial and variable and have been the subject of studies, but a range between 100 and 286 N [21] for anterior regions and 250 and 392 N [17] for posterior regions are considered. This variation occurs due to different techniques and materials used to measure this load; there is no standardization of these techniques, materials, and no consensus among researchers [22]. In the current study, as shown in Table 2, the three connections tested showed excellent results, with no mechanical failures; only the two-piece abutments of the 11.5° connection (G3 and G4) showed values below what the literature reports [17]; however, the tests show variations [21], and in the mouth we would hardly have an occlusal load in 30°.

The G4 implant-abutment set showed the lowest load values ​(360.88 N) after dynamic loading but has greater reversibility, while the G6 one-piece abutment showed greater resistance (408.65 N) but does not show the same reversibility. Observing that the two abutments were tested on the same implant (11.5°), it leads to believe that the selection of the abutment is clinically important considering mechanical resistance and reversibility. However, the G2 two-piece abutment would be the most suitable for all regions of the mouth, as this abutment has greater reversibility compared to one-piece and greater resistance (404.57 N) than the G4 two-piece abutment.

The tested implants and abutments have different manufacturing characteristics; the G1 and G2 abutments have a component self-removal system after the screw is loosened, and the G3 and G4 abutments that showed the worst results may suffer frictional locked at the implant-abutment junction; therefore, it may be interesting in future studies to carry out tests of loosening of screws and pull-out to assess the mechanical stability of the connections. Clinical studies could also test the stability of these connections, taking measurements of the bone level and assessing the success rate and survival of the prostheses in anterior and posterior teeth.

## Conclusions

In conclusion, the maximum force supported was influenced by oblique cyclic loading in the implant-abutment assemblies of the G4 and G6 but was not influenced in the G2. Clinically, the G2 connection has good mechanical resistance and reversibility, being more suitable for cemented single rehabilitation.

## Data Availability

The datasets generated and analyzed during the current study are available from the corresponding author upon reasonable request.

## Abbreviations

G1: Group 1
G2: Group 2
G3: Group 3
G4: Group 4
G5: Group 5
G6: Group 6
GM: Grand Morse
CM: Morse taper
PR: Parana
Ø: Diameter
Fle: Flexural elastic limit
